# Biomechanics for Orthodontic Intrusion of Severely Extruded Maxillary Molars for Functional Prosthetic Rehabilitation

**DOI:** 10.1155/2019/8246129

**Published:** 2019-11-15

**Authors:** Ivan Pedro Taffarel, Thiago Martins Meira, Lara Karolina Guimarães, Oscar Mario Antelo, Orlando Motohiro Tanaka

**Affiliations:** ^1^School of Life Sciences, Pontifícia Universidade Católica do Paraná, Curitiba, Brazil; ^2^Brazilian Board of Orthodontics and Dentofacial Orthopedics, Brazil; ^3^Bahia State University (UNEB), Guanambi, Bahia, Brazil; ^4^Universidad Intercontinental, Cidade de Mexico, Mexico; ^5^The Center for Advanced Dental Education, Saint Louis University, USA

## Abstract

The objective of this clinical case is thus to present a Class II, division 1, subdivision malocclusion with a severely extruded maxillary left hemiarch, which, due to the loss of mandibular teeth, makes prosthetic rehabilitation of the edentulous spaces impossible. A significant intrusion was performed with mini-implants followed by miniplates associated with fixed appliance and elastomeric chains. The results of this process showed that the biological responses of the teeth and the surrounding bony structure to the intrusion were demonstrated to be normal and acceptable. A clinically significant intrusion of the left maxillary molars, along with the recovery of the interocclusal space and the prosthetic rehabilitation, was obtained with a fixed orthodontic appliance that was associated to the biomechanics with TADs. It also allowed the obtaining of Class I canine relationship, demonstrated periodontal health and favored the prosthetic rehabilitation with good occlusion, aesthetics, and satisfactory function.

## 1. Introduction

Posterior teeth that are supraerupted due to the early loss of their antagonists are commonly seen in adults that have limited or no access to dentistry during childhood and adolescence [1]. An early loss of any molar is bound to cause supraeruption of the opposing molar into the available space. Overeruption of such a molar can lead to occlusal interference and functional disturbances and cause great difficulty during prosthetic reconstruction [2].

Orthodontic treatment of overerupted molars has always been considered challenging for most orthodontists, as it is one of the most difficult movements to achieve during orthodontic treatment [3]. This is primarily due to the greater root volume of these teeth [4]. Pure intrusion can only be achieved when an adequate anchorage system is available to support the light and continuous forces that are directed through the tooth's center of resistance [5].

Various approaches have been proposed to intrude overerupted molars, including the use of removable appliances with elastics [6], modified palatal arches [7], elastomeric chains [8], magnets [9], and skeletal anchorage systems [10].

Currently, temporary anchorage devices (TADs), such as mini-implants and miniplates, are the treatment of choice for enhancing orthodontic tooth movement. These devices promote absolute anchorage, and the teeth can be moved immediately after the placement, with no need for patient collaboration. TADs reduce the orthodontic treatment period, minimize the discomfort during treatment, favor aesthetics, and increase the predictability of the final result.

The purpose of this clinical report is to describe a case of Class II, division 1, subdivision malocclusion that had severe maxillary left molar extrusion, and the direct use of mini-implants and miniplate for maxillary molar intrusion was chosen to create the necessary space for prosthetic rehabilitation.

## 2. Case Report

### 2.1. Diagnosis and Etiology

The patient was a 26.2-year-old female, who had presented for an initial consultation at the orthodontic office with the chief complaint that “the top teeth have gone down.”

**Figure 1:**
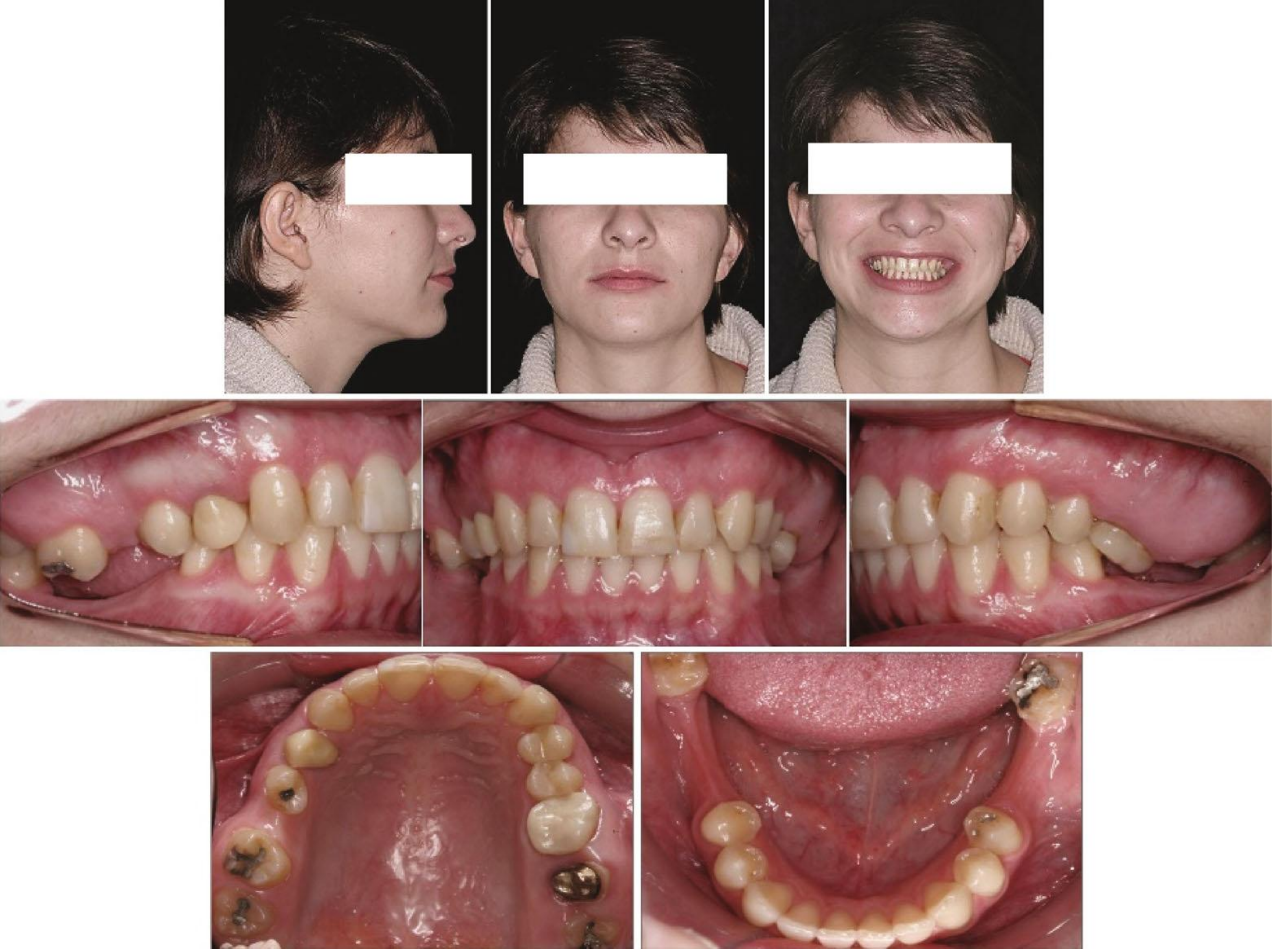
Pretreatment facial and intraoral photographs.

**Figure 2:**
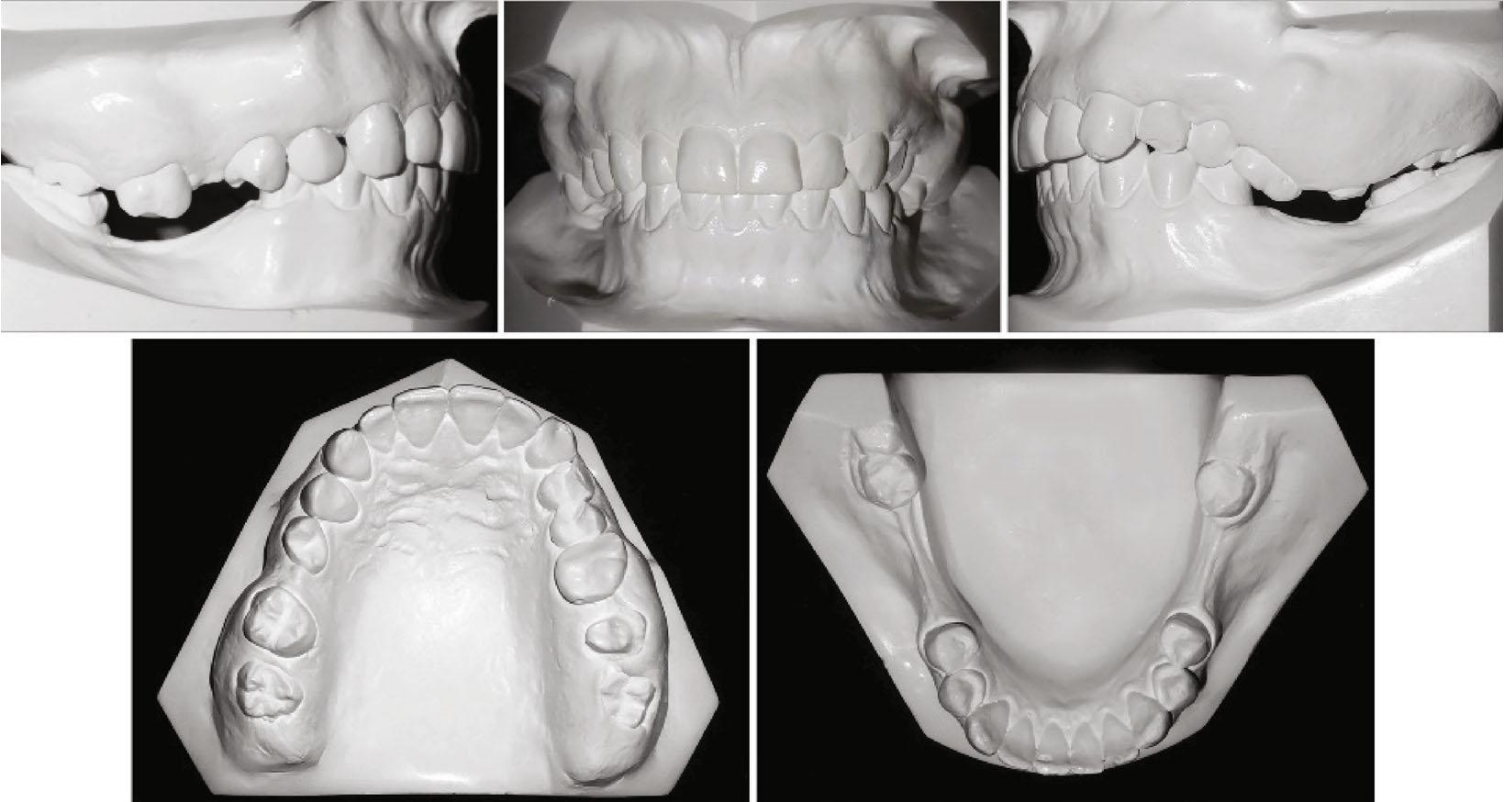
Pretreatment dental casts.

In the extraoral examination, she presented a concave profile and an asymmetric smile. Clinically presented, she had a Class II, division 1, subdivision malocclusion (4.0 mm), overjet, moderate overbite, and an absence of the first and second mandibular molars and maxillary right first molar. With severe extrusion of the maxillary molars, more severe on the left side, the maxillary midline deviated 2.0 mm to the right, and the mandibular deviated 1.0 mm to the left. (Figures 1 and 2).

**Figure 3:**
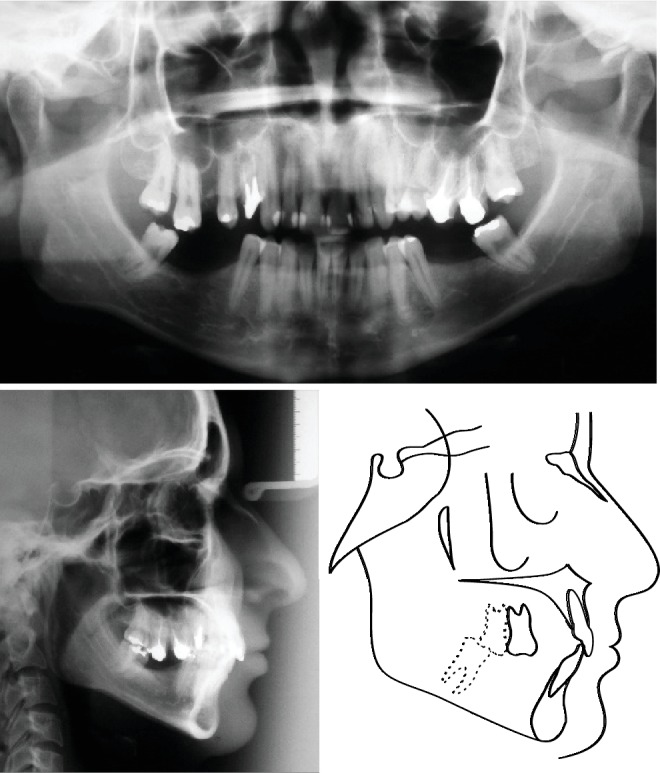
Pretreatment panoramic radiograph, lateral cephalometric radiograph, and tracing.

In the radiographic examination, it was verified that there was an impacted maxillary left third molar and endodontic treatment and intra-radicular metal pins in maxillary right first premolar, left maxillary first, and second molars. Both mandibular third molars were mesially tilted, and the maxillary incisor had big restorations. (Figure 3). Cephalometric radiograph presented a skeletal Class I (ANB = 2°), upright maxillary incisors and slightly proclined mandibular incisors (Table 1).

### 2.2. Treatment Objectives

The treatment objective of this patient was thus to correct Class II on the left side, attain an ideal overjet and overbite, correct the midline deviation, intrude maxillary posterior teeth, and recover space for prosthetic rehabilitation. This was in addition to maintaining the optimal facial balance and aesthetics.

### 2.3. Treatment Alternatives

Alternative treatments are as follows:

1. Reduction of the crown height of the second right maxillary molar or an extraction of the maxillary left first, second, and third molars to create space surgically for prosthesis rehabilitation
2. An intrusion of the maxillary molars with the fixed appliance of the maxillary molars in both sides, using TADs
3. Intruding maxillary molars with the fixed appliance of the maxillary molars in both sides with corticotomy, removing some piece of the alveolar bone to make adequate space for prosthesis rehabilitation on the left side
4. Surgically leveling both intruding maxillary posterior teeth
5. Surgically leveling the intruding maxillary left posterior teeth and intruding the right maxillary molar with TADs

In the mandibular arch, the third molars were rectified by making them upright, along with prosthesis rehabilitation in both sides.

### 2.4. Treatment Progress

**Figure 4:**
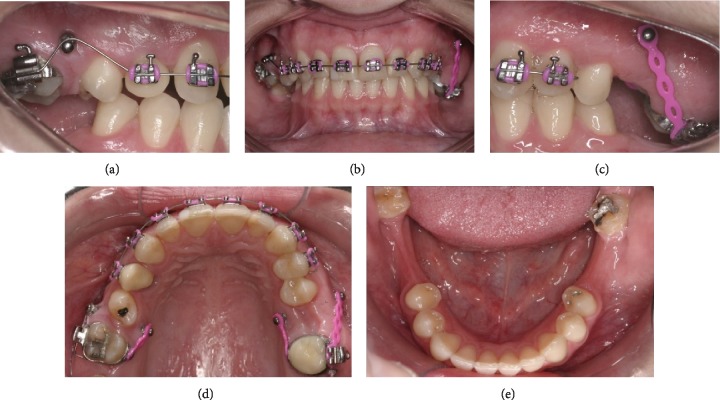
Progress starting the intrusion of maxillary left molars.

Initially, Roth .022‐in × .028‐in prescription brackets were bonded only to the maxillary arch (Figure 3(a)), and after starting the alignment and leveling stages with a .018-in SS archwire, four mini-implants were installed, one buccal and one palatal (Figures 4(a), 4(c), and 4(d)), with the objective of absolute anchorage for posterior tooth intrusion and Class II correction on the left side and a correction of the maxillary midline deviation.

**Figure 5:**
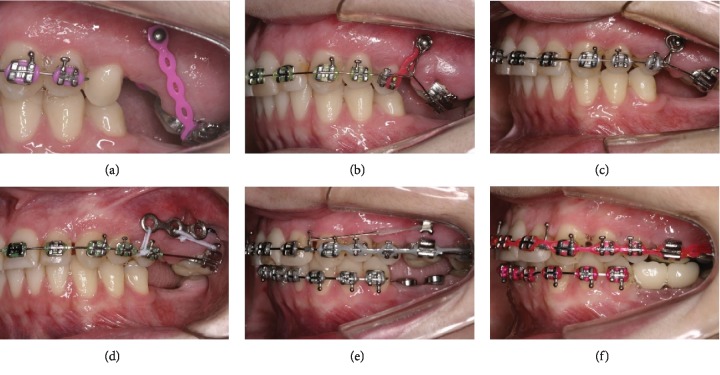
Progress sequence of the intrusion of maxillary left molars from first to finishing stages.

The mini-implants on the left side as adjunctive for the intrusion also facilitated the distalization and thus corrected Class II on this side. After a year of treatment, brackets were bonded into the mandibular arch. In spite of the good evolution, there was a reduction in the vertical distance of the maxillary left posterior teeth in relation to the mini-implant, thus making it necessary to replace the buccal mini-implant with a miniplate (Figures 5(a)–5(c)).

The intrusion of both molars was achieved by using a combination of a mini-implant, TMA spring, and elastic chains. NiTi springs were also used for the anterior retraction and thus facilitated the correction of the Class II dental relation and the maxillary midline deviation (Figures 5(d)–5(f)).

**Figure 6:**
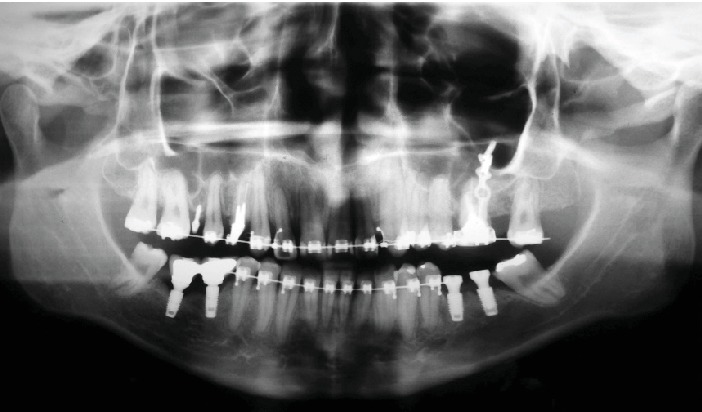
Progress panoramic radiograph.

The mandibular third molars were not considered as their extractions were programmed. Prior to the removal of the maxillary and mandibular fixed appliances, temporary acrylic crowns were affixed over the lower implants, to maintain the recovered vertical dimension (Figure 6).

### 2.5. Treatment Results

**Figure 7:**
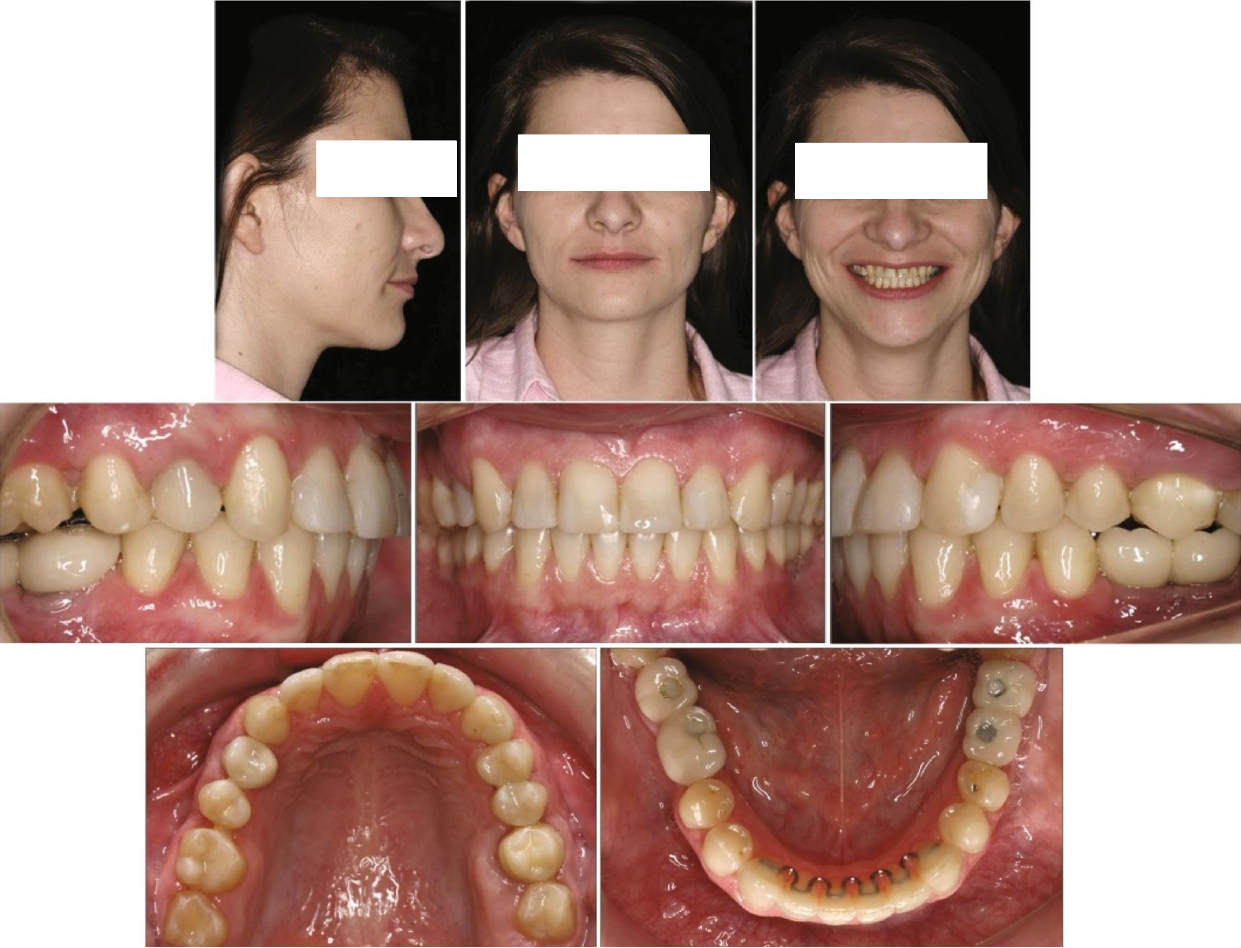
Posttreatment facial and intraoral photograph.

**Figure 8:**
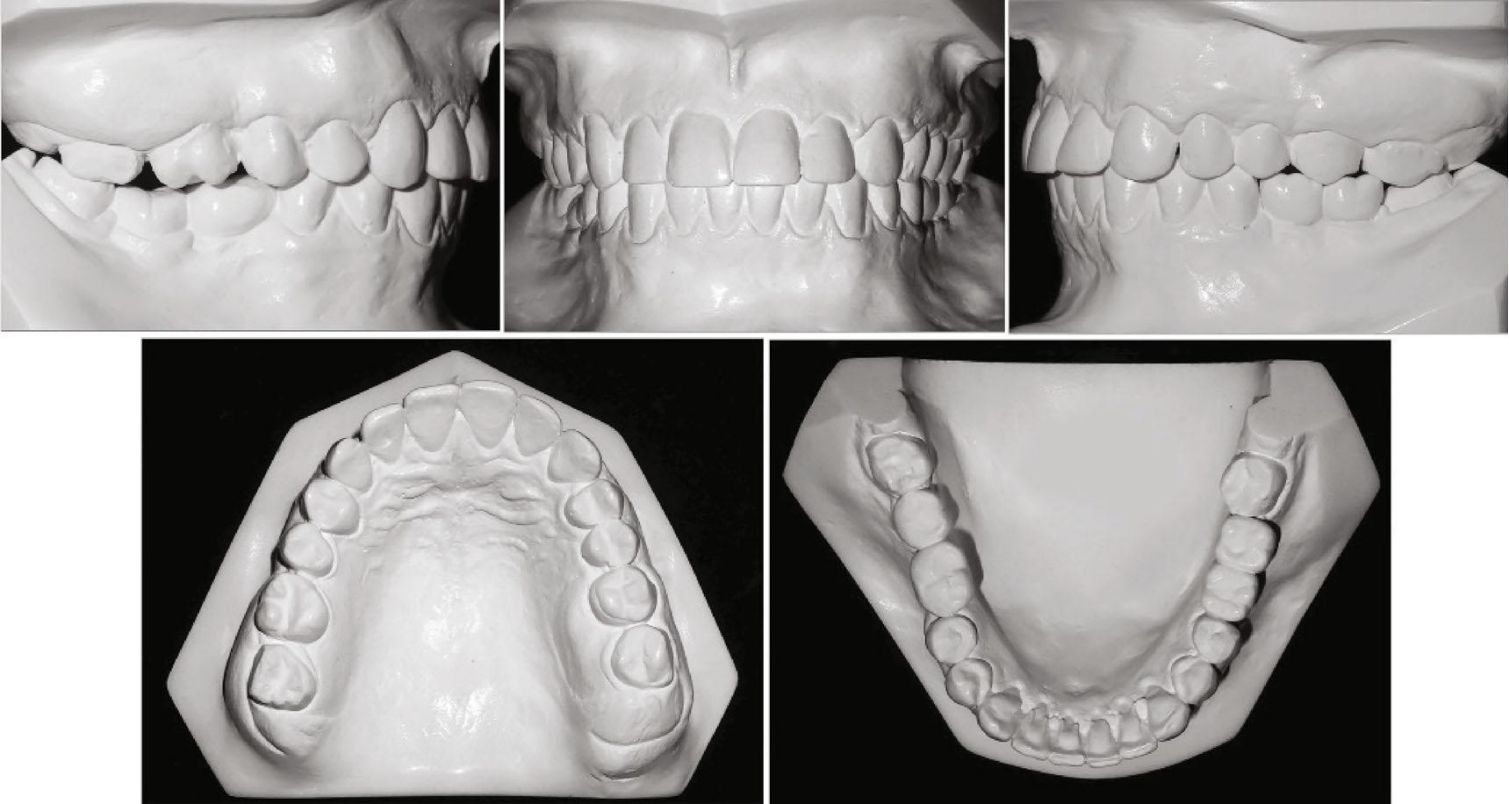
Posttreatment dental casts.

Facially, her smile became symmetrical, balanced, and harmonious (Figure 7, facial). After the correction of the Class II relationship, the maxillary midline deviation, maintenance of the overjet and overbite, and after the recovery of the prosthetic space, a new alignment and leveling, along with subsequent intercuspation and finalization phases, were performed. After the removal of the appliance, a fixed canine-to-canine retention (0.6 mm), was bonded, and a wraparound-type removable appliance were used for the full duration of a year, after which it was used for another year, but only at night during sleeping hours (Figures 7 and 8).

**Figure 9:**
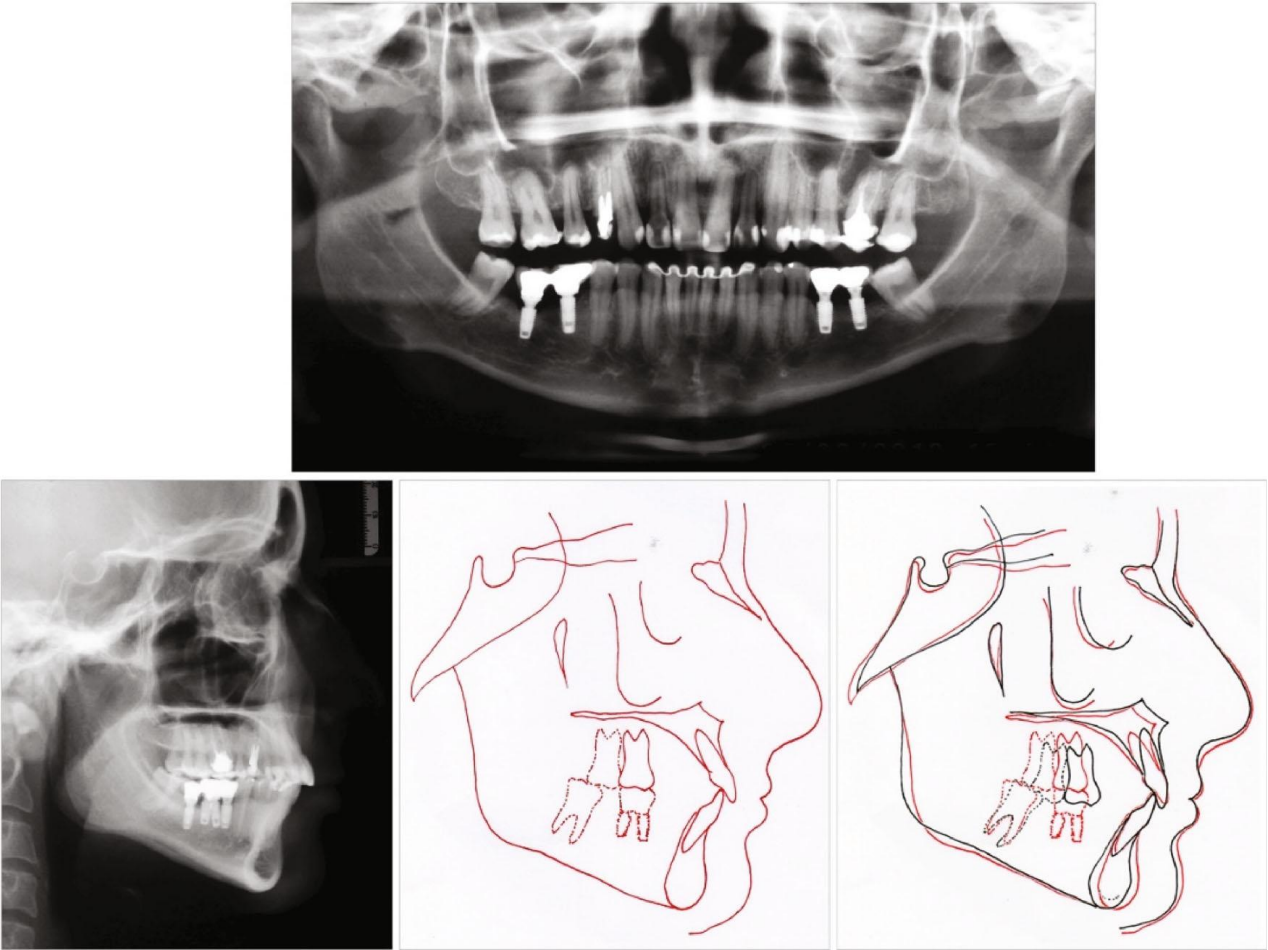
Posttreatment panoramic radiograph, lateral radiograph, lateral cephalometric radiograph tracing, and superimposition: before treatment (black) and after treatment (red).

The panoramic radiograph shows that the maxillary left molars' alveolar bone level is in good shape, reasonable root parallelism, and implant-prosthetic rehabilitation in the mandibular arch (Figure 9). The cephalometric measurements and superimposition revealed a proclination of the maxillary incisors and the maintenance of a good profile line (Figure 9, Table 1).

**Figure 10:**
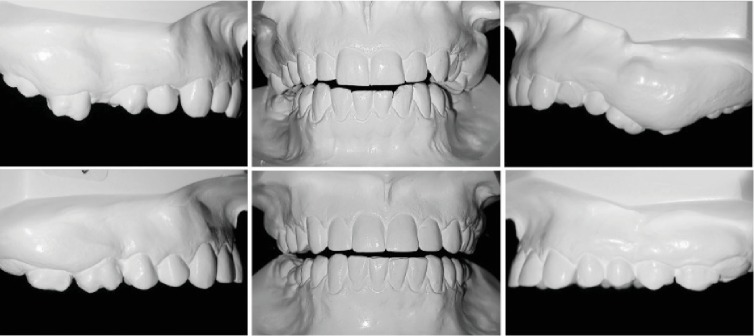
Pretreatment and posttreatment dental casts. Uneven occlusal plane.

The occlusal line was leveled with the isolated intrusion of maxillary right second molar and the intrusion of both left molars, combined with alveolar bone upward movement (Figure 10).

## 3. Discussion

This patient's treatment demonstrated the efficacy of the direct use of orthodontic mini-implants for the correction of extruded, maxillary first molars.

It is common for adult patients with dental loss, particularly of molars and premolars, to have an extrusion of the antagonist, thus rendering prosthetic rehabilitation difficult [11]. In these cases, the use of TADs along with orthodontic biomechanics is used to obtain better case control while minimizing unwanted side effects [9, 11, 12], as described in the present case report in an interdisciplinary approach.

When prosthodontic treatment of a missing molar has been delayed, the traditional treatment has been used to reduce the crown length of the tooth opposite the extruded tooth [13], or to adjust the path of intrusion. Intrusion by subapical osteotomy [14] and extraction of the extruded molar are more aggressive alternatives, but most patients today refuse to sacrifice a healthy tooth.

Many patients often decline the latter option and would prefer a restoration of the mandibular occlusion. Depending on the patient's cooperation, the treatment would include either a shortening of the arch length or the selection of a treatment plan with extensive reduction of the supererupted maxillary molar, thus requiring endodontic treatment, periodontal surgery, and crown restoration, or even surgical impaction of the extruded teeth [4] as presented in the present clinical case.

In this reported clinical case, the first step was the use of mini-implants. Two mini-implants were installed on each side, one buccally and another palatally, to have more controlled movement and to make it less complex for the professional, with more predictable results [15]. For pure intrusion, a total of three mini-implants could be used in a tooth, in agreement with Paccini et al. [16]. Alternatively, a combination of selective alveolar corticotomies with a full-coverage splint which is modified to incorporate superelastic NiTi coil springs [1] can be viable in efficiently intruding the overerupted maxillary molars and reduce surgical risks, treatment time, and costs for both orthodontists.

To avoid root resorption, intrusive force levels should be kept optimal. While optimal force has not yet been suggested for intrusion with miniscrews, forces greater than what is generally accepted for intrusion in conventional treatments are reported to be applied with miniscrews and miniplates [17]. In the present clinical case, light forces of approximately 50 g were used in each molar, as reported by Melsen et al. [5], although some authors suggest intrusive forces of 150 g [11] and 100 g [18].

Although our clinical case has presented a good evolution with TADs, it was necessary to replace the mini-implant more cervical. However, it would imply positioning it in the alveolar mucosa, which would be contraindicated. Therefore, a miniplate was chosen for the left side, where the extrusion was larger and due to the little space between the roots of the posterior for the mini-implant, to further continue the movement of intrusion and to increase the vertical distance with the tooth antagonists.

In a finite element study, a unilateral force promoted higher stress in the root apex and higher dental tipping, and the bilateral forces promoted better distribution without evidence of dental tipping. However, the bilateral intrusion technique suggested a lower probability of root apex resorption [19], as applied in the present case.

There was no clockwise rotation of the mandible, which is a commonly seen side effect in the correction of anterior open bite. This is different from the present case report as there was an absence of teeth in the opposite arch. The periapical radiographs showed improvement in the crown-to-root ratios, no adjacent tooth extrusion, and no root resorption, which was in agreement with the results obtained by Oliveira et al. [1].

The increasing demands by adult patients for interdisciplinary dental treatment, in general, often lead to a need for methods with minimal complications, as well as rapid and comfortable solutions that require no tooth support, no aesthetic compromise, and minimal patient compliance. As such, mini-implants and miniplates have been found to be an effective method of treatment for molar intrusion, with relatively simple installation and removal.

These devices have been used in the orthodontic office with increasing frequency, particularly in cases where an inadequate number of dental units stand in the way of an effective anchorage, or even when only used to simplify orthodontic mechanics and make it more predictable. Hence, in the present clinical case, TADs were used to make molar intrusion possible in this case, which involved extremely overerupted maxillary left molars.

## 4. Conclusion

A clinically significant intrusion of the left maxillary molars, with the recovery of the interocclusal space and prosthetic rehabilitation, was obtained with the fixed orthodontic appliance associated with the biomechanics with TADs. A Class I canine relationship was also obtained, with a correction of midline deviation and a restoration of occlusion with implants, along with good occlusion, aesthetics, and satisfactory function.

## Figures and Tables

**Table 1:** Cephalometric measurements.

Measurements	Norms	Pretreatment	Posttreatment
SNA angle (°)	82	89	88
SNB angle (°)	80	84	85
ANB angle (°)	2	5	3
Ao-Bo (mm)	♀ 0 ± 2 ♂ 1 ± 2	-3	0
Facial angle (°)	87	87	88
Convexity (°)	0	13	88
FMA (°)	25	21	20
GoGn-SN (°)	32	27	23
*Y*-axis (°)	59	61	59
1-NA (mm)	4	3	7
1-NA (°)	22	13	28
1-NB (mm)	4	7	6
1-NB (°)	25	30	29
IMPA (°)	90	97	101
Interincisal angle (°)	132	134	121
*Z* angle (°)	75	78	77

Follow up.
